# Evaluation of a Magnetic Compression Anastomosis for Jejunoileal Partial Diversion in Rhesus Macaques

**DOI:** 10.1007/s11695-023-07012-4

**Published:** 2023-12-23

**Authors:** Lauren L. Evans, William G. Lee, Mohammad Karimzada, Veeshal H. Patel, Vamsi K. Aribindi, Dillon Kwiat, James L. Graham, David E. Cummings, Peter J. Havel, Michael R. Harrison

**Affiliations:** 1 Department of Surgery, University of California San Francisco, San Francisco, USA; 2 Department of Molecular Biosciences, School of Veterinary Medicine and Department of Nutrition, University of California Davis, Davis, USA; 3 Division of Metabolism, Endocrinology and Nutrition, University of Washington and VA Puget Sound Health Care System, Seattle, USA

**Keywords:** Insulin resistance, Metabolic surgery, Staple-free anastomosis, Magnetic compression device, Magnamosis, Small-bowel anastomosis

## Abstract

**Purpose:**

Metabolic surgery remains underutilized for treating type 2 diabetes, as less invasive alternative interventions with improved risk profiles are needed. We conducted a pilot study to evaluate the feasibility of a novel magnetic compression device to create a patent limited caliber side-to-side jejunoileal partial diversion in a nonhuman primate model.

**Materials and Methods:**

Using an established nonhuman primate model of diet-induced insulin resistance, a magnetic compression device was used to create a side-to-side jejunoileal anastomosis. Primary outcomes evaluated feasibility (e.g., device mating and anastomosis patency) and safety (e.g., device-related complications). Secondary outcomes evaluated the device’s ability to produce metabolic changes associated with jejunoileal partial diversion (e.g., homeostatic model assessment of insulin resistance [HOMA-IR] and body weight).

**Results:**

Device mating, spontaneous detachment, and excretion occurred in all animals (*n* = 5). There were no device-related adverse events. Upon completion of the study, ex vivo anastomoses were widely patent with healthy mucosa and no evidence of stricture. At 6 weeks post-device placement, HOMA-IR improved to below baseline values (*p* < 0.05). Total weight also decreased in a linear fashion (*R*^2^ = 0.97) with total weight loss at 6 weeks post-device placement of 14.4% (*p* < 0.05).

**Conclusion:**

The use of this novel magnetic compression device to create a limited caliber side-to-side jejunoileal anastomosis is safe and likely feasible in a nonhuman primate model. The observed glucoregulatory and metabolic effects of a partial jejunoileal bypass with this device warrant further investigation to validate the long-term glucometabolic impact of this approach.

**Graphical Abstract:**

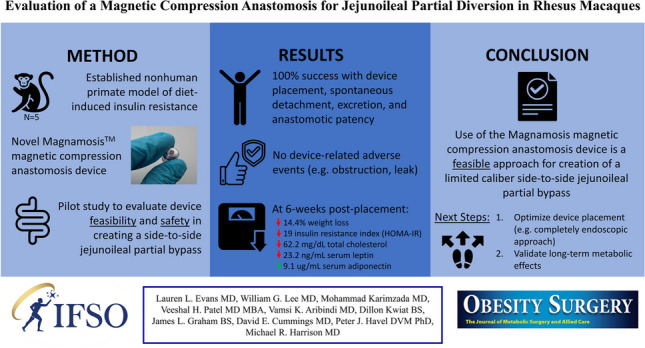

**Supplementary Information:**

The online version contains supplementary material available at 10.1007/s11695-023-07012-4.

## Introduction

Type 2 diabetes (T2DM) affects more than 400 million people worldwide, with an estimated annual healthcare cost of $327 billion [1, 2]. Effective and durable therapies for T2DM remain elusive, and identifying more effective interventions to treat T2DM is a top priority in medicine [1]. Our growing experience with metabolic-bariatric surgical procedures has revealed that surgical reconfiguration of the gastrointestinal (GI) tract can exert powerful corrective effects on glucose homeostasis, independent of weight loss [3–5]. A dozen randomized clinical trials have confirmed that metabolic surgery results in superior long-term glycemic control and remission of T2DM compared to pharmacotherapy and lifestyle modification [6–8]. As of 2016, there has been international consensus that metabolic surgery should be included among standard T2DM treatment options [8]. However, the risks of major short- and long-term complications with conventional metabolic surgeries continue to limit adoption [9–13]. Less invasive alternative interventions with more favorable risk profiles are needed [14].

Innovative surgical interventions have the potential to produce highly favorable metabolic results through minimally invasive techniques, by augmenting the gut hormone milieu in a targeted fashion. Limited reconfiguration of the GI tract, such as the jejunoileal partial bypass, is designed to allow a portion of enteric contents to bypass the majority of the small intestine while also allowing for nutrient flow through the bypassed segment of small bowel. As a result, this bypass exploits the primary drivers behind the glucoregulatory effects of conventional metabolic surgery which can result in robust metabolic benefits, while nutrient flow through the bypassed segment may avoid the undesired adverse sequelae associated with the more extensive surgical GI reconstruction of traditional metabolic surgery (e.g., malnutrition sequelae, bacterial overgrowth, and cirrhosis) [15]. However, prior preclinical and in-human studies evaluating magnetic compression devices in this context remain limited, and no prior studies have investigated the use of the Magnamosis™ magnetic compression device for this approach [15, 16].

This pilot study aimed to evaluate the feasibility of creating a side-to-side jejunoileal anastomosis using the Magnamosis™ magnetic compression device to create a patent limited caliber jejunoileal partial bypass in a nonhuman primate model. Our primary aims were to demonstrate feasibility and safety with Magnamosis device use while creating a functional jejunoileal side-to-side anastomosis. Our secondary aim was to evaluate the metabolic effects of the limited caliber jejunoileal partial bypass created with the Magnamosis device in an established rhesus macaque diet-induced insulin resistance (IR) model.

## Materials and Methods

### Study Design

We conducted a preclinical pilot study evaluating the feasibility of creating a side-to-side jejunoileal anastomosis with a magnetic compression device as a minimally invasive alternative to conventional metabolic surgery. The metabolic effects of the resulting limited-caliber jejunoileal partial diversion were evaluated in a nonhuman primate model of diet-induced IR, analogous to the IR to T2DM disease process described in humans [17–19]. Five adult male rhesus macaques (*Macaca mulatta*), age 12–20 years (mean initial body weight 17.9 kg ± 1.2 kg), were selected for inclusion after laboratory screening confirmed the absence of pre-existing IR or T2DM. Primary outcomes evaluated feasibility (device mating, device detachment/excretion, and anastomosis patency) and safety (any device-related complications, anastomotic leak, and stricture). Secondary outcomes evaluated the device’s ability to produce metabolic changes associated with jejunoileal partial diversion such as weight loss, the homeostatic model assessment of insulin resistance (HOMA-IR), and serum metabolic markers at 3 and 6 weeks post-intervention (lipids, adiponectin, leptin, and glucagon-like peptide-1 (GLP-1)). At 6 weeks, necropsy with en bloc resection of the jejunoileal anastomosis was performed.

IR was induced using the protocol for an established model of nonhuman primate diet-induced IR [17]. A high-sugar diet (75 g fructose/day) was initiated 8 weeks prior to intervention and continued after intervention for the duration of the study period.

The California National Primate Research Center provided and maintained the rhesus macaque animals for this study. Animals were housed individually and provided environmental enrichment with food and water access. Animals were euthanized (sodium phenobarbital, 120 mg/kg intravenous (IV)) in accordance with California National Primate Research Center standard operating procedures LL-08 and LL-02. Institutional approval of the study protocol was obtained from the University of California Davis Institutional Animal Care and Use Committee (protocol 17,686), and experiments were conducted per the United States Department of Agriculture Animal Welfare Act and the National Institute for Health’s *Guide for the Care and Use of Laboratory Animals (NIH Publications No. 8023, revised 1978)*. Study findings are reported following the ARRIVE 2.0 guidelines.

### Magnetic Compression Anastomosis

**Fig. 1:**
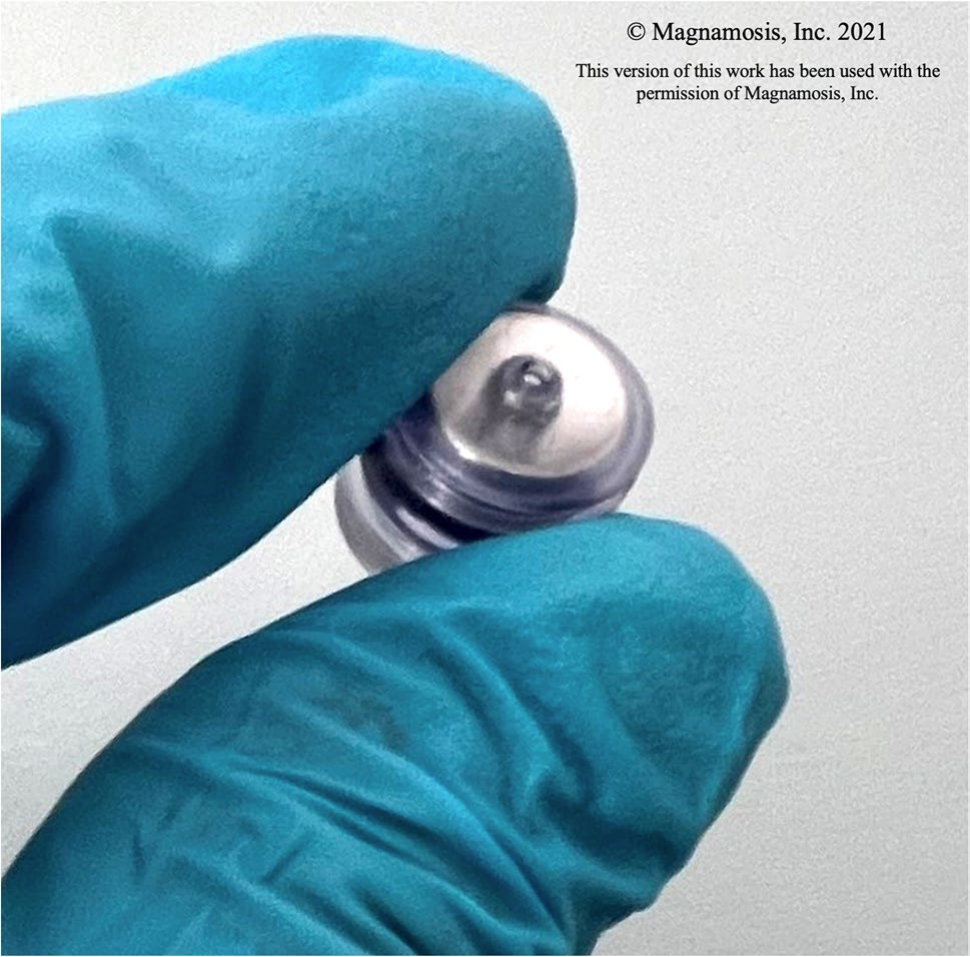
The Magnamosis device ((cc) Magnamosis, Inc. 2021; San Francisco, CA, USA)

A Magnamosis magnetic compression device (15.5-mm outer diameter) was used to create a side-to-side jejunoileal anastomosis. Magnetic compression is an emerging technology for creating GI anastomoses without sutures or staples. The Magnamosis family of devices (Magnamosis, Inc.; San Francisco, CA, USA) employs a pair of magnetic anchors amenable to endoscopic delivery to create a GI anastomosis. The Magnamosis device system includes two ring-shaped magnetic anchors, each composed of a rare-earth magnetic core (neodymium-iron-boron) in a polycarbonate shell (Fig. 1). This device has a compressive force of *F*_R3_ = 2.2 N at a reference 3-mm face separation, corresponding to a spatially averaged pressure on the interposed double-layer of intestinal tissue of *P*_R3_ = 14 kPa. Selection of this device’s force and pressure ratings was grounded in extensive work in animal models focused around safety and mechanical robustness of GI tract anastomosis creation [20, 21]. This magnetic compression device is designed to create an anastomosis over 7–14 days, after which the mated anchor pair subsequently spontaneously detach from the physiologically mature anastomosis to pass in the fecal stream.

### Procedural Approach

Before each intervention, animals were prepared in a sterile operating room and placed under anesthesia according to California National Primate Research Center standard operating procedures II-01 and II-02. Interventions were performed by the study team with assistance from California National Primate Research Center veterinary staff.

### Anesthesia Protocol

Animals underwent induction and intubation by veterinary staff. Anesthesia was induced with inhaled isoflurane (1.5–2% titrated to effect) and maintained with ketamine (0.25–1.5 mg/kg/min IV). Depth of anesthesia was monitored with multi-modal cardiorespiratory monitoring, including pulse oximetry, capnography, electrocardiography, blood pressure, pulse rate, and reflexes.

### Surgical Procedure

After general anesthesia was induced, a midline laparotomy was performed. A 15.5-mm magnetic anchor was placed in each the proximal jejunum and ileum. The proximal anchor was delivered to the proximal jejunum via upper endoscopy and then manually positioned approximately 20 cm (± 10 cm) distal to the ligament of Treitz. The distal anchor was placed through an 8-mm mid-jejunal enterotomy and manually maneuvered until approximately 20 cm (± 10 cm) proximal to the ileocecal valve. The enterotomy was closed primarily. The proximal jejunum and distal ileum were brought into proximity, and the anchors were mated magnetically at the antimesenteric border. The bowel was returned to the abdomen, and the abdomen was irrigated. The abdominal wall was closed primarily in layers. Animals were recovered and monitored according to a standard veterinary protocol.

### Post-procedure Evaluation

Post-operative analgesia (oxymorphone 0.15 mg/kg IM, buprenorphine 0.01–0.03 mg/kg IM) was continued for a minimum of 3 days. Soft food was offered 12 h after intervention, and diet was advanced ad libitum. Blood glucose was monitored daily for a minimum of 2 weeks. Meal completion and liquid oral intake were monitored. Animals were evaluated for signs of rapid weight loss, malabsorption, and malnutrition. Weight was obtained weekly. Cages and stool were regularly assessed for passage of the mated anchor pair. An abdominal x-ray was obtained if the anchors had not passed 2 weeks post-intervention.

### Laboratory Monitoring

Blood samples were collected before initiating the high-sugar diet, 2 days before surgical intervention, and at 3 and 6 weeks post-intervention. Animals were fasted overnight for no longer than 16 h before sampling. Measurement of all biochemical parameters was completed at the University of California Davis, Department Nutrition Assay Services Unit. Plasma insulin concentrations were quantified with a radioimmunoassay (Millipore, St. Charles, MO, USA), and plasma glucose concentrations were determined using a YSI Glucose Analyzer (YSI Life Sciences, Yellow Springs, OH, USA). Plasma lipids were measured using a Polychem Chemistry Analyzer (PolyMedCo, Inc. Cortlandt Manor, NY, USA). Plasma leptin and adiponectin concentrations were assessed by radioimmunoassay (Millipore, St. Charles, MO, USA). Plasma concentrations of active GLP-1 were quantified with an electro-chemiluminescent immunoassay (Meso Scale Discovery, Rockville, MD, USA).

HOMA-IR assessed systemic IR and was calculated using the equation [fasting glucose (mmol/L) × fasting insulin (µU/mL)]/22.5 [22].

### Statistical Methods

Data are reported as mean ± standard error of the mean (SEM) or median ± interquartile range (IQR). Statistical significance was determined using Friedman’s test. *p* value < 0.05 was considered statistically significant. Analysis was performed using MATLAB software package (Mathworks, Natick, Massachusetts, USA).

## Results

### Diet-Induced Insulin Resistance

Fasting plasma insulin and HOMA-IR increased by greater than twofold after 8 weeks on the high-sugar diet without significant change in fasting plasma glucose, indicating the presence of IR without overt T2DM [23]. Metabolic parameters before and after the high-sugar diet are summarized in Supplemental Table 1.

### Device-Related Outcomes

Device placement with complete mating occurred in all monkeys (*n* = 5). Postoperatively, all monkeys tolerated diet advancement and maintained adequate oral intake. The mated anchors passed spontaneously without complication in all animals within 2 weeks of intervention, and no follow-up x-rays were required. There were no device-related complications, including intestinal obstruction or anastomotic leak.

Gross inspection of all five resected side-to-side jejunoileal anastomoses revealed healthy-appearing bowel and mesentery. The serosal surface of all anastomoses appeared well healed. All anastomoses were widely patent. The mucosa of all anastomoses appeared healthy and well healed, without evidence of inflammation or stricture.

### Metabolic Outcomes

Changes in metabolic outcomes immediately before device placement, then at 3 and 6 weeks post-intervention, are summarized in Supplemental Table 2.

**Fig. 2:**
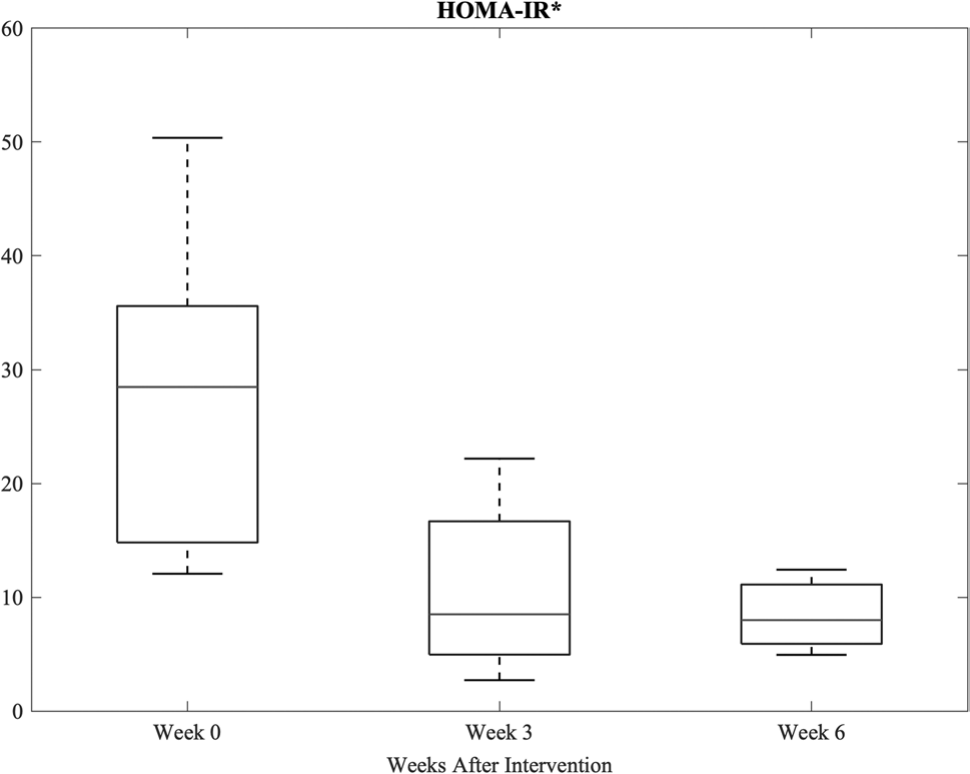
HOMA-IR 3 and 6 weeks after intervention compared to pre-intervention baseline (week 0). Data are reported as median (IQR). *Statistically significant change from baseline (*p* < 0.05). HOMA-IR, homeostatic model assessment of insulin resistance

HOMA-IR (percent change 59.0–84.1%, *p* < 0.05, Fig. 2) and fasting plasma insulin (percent change 53.8–71.8%, *p* < 0.05, Supplemental Table 2) decreased significantly, reaching values below pre-diet baseline. There was no significant change in fasting plasma glucose concentrations (Supplemental Table 2).

**Fig. 3:**
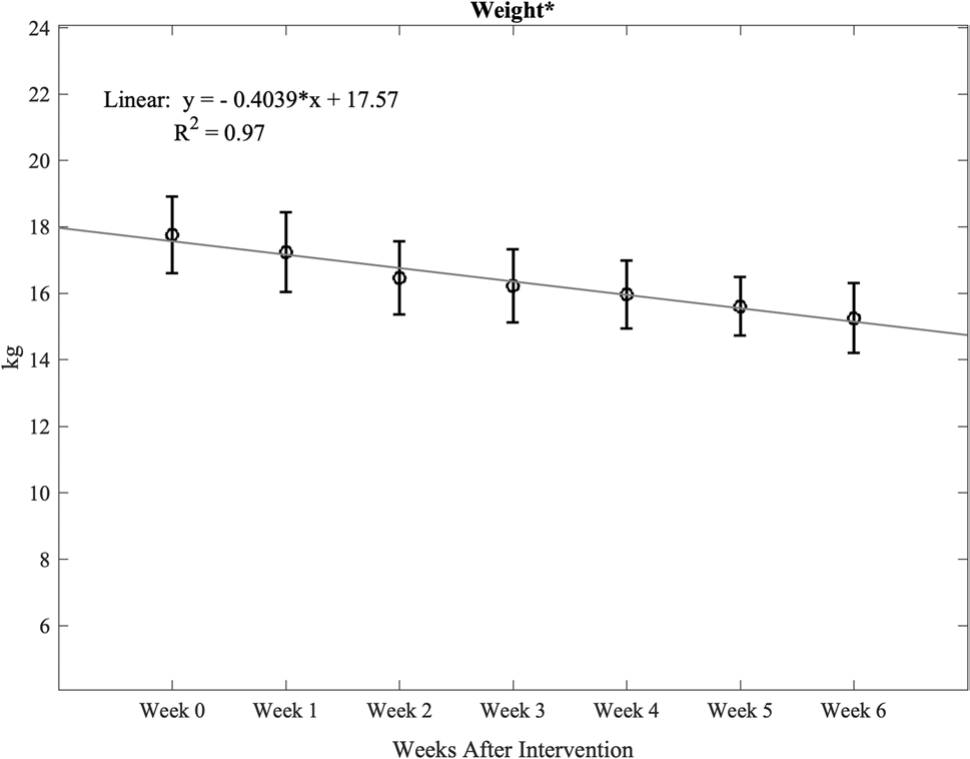
Weight loss from time of intervention (week 0) to 6 weeks. A strong linear decrease (*R*.^2^ = 0.97) results in a 14.4% fall in total body weight at 6 weeks (*p* = 0.004). Data are reported as mean (SEM). *Statistically significant change from baseline (*p* < 0.05)

**Fig. 4:**
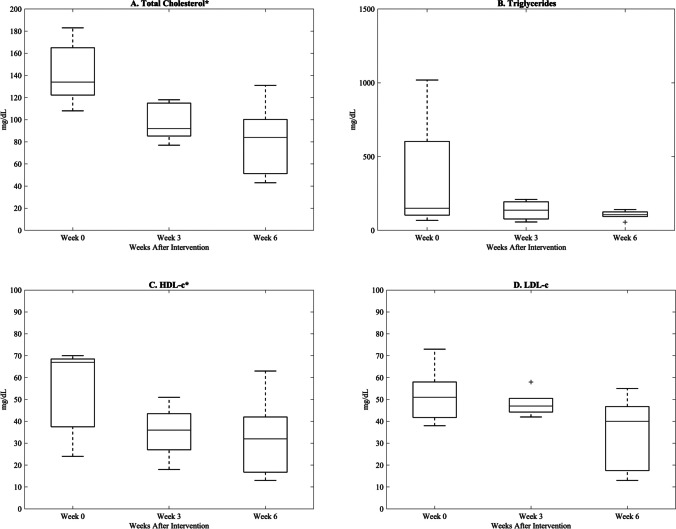
Fasting plasma concentrations of total cholesterol (**A**), triglycerides (**B**), HDL-c (**C**), and LDL-c (**D**) at 3 and 6 weeks after intervention compared to pre-intervention baseline (week 0). Data are reported as median (IQR). *Statistically significant change from baseline (*p* < 0.05). LDL-c, low-density lipoprotein cholesterol; HLD-c, high-density lipoprotein cholesterol

**Fig. 5:**
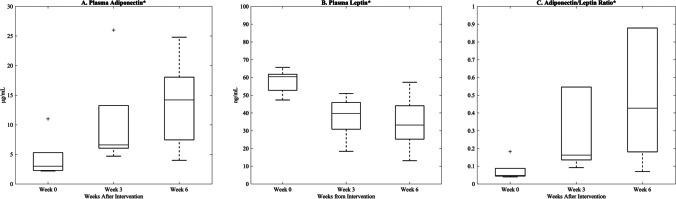
Plasma concentrations of adiponectin (**A**), leptin (**B**), and the adiponectin/leptin ratio (**C**) at 3 and 6 weeks after intervention compared to pre-intervention baseline (week 0). Data are reported as median (IQR). *Statistically significant change from baseline (*p* < 0.05)

Weight loss occurred in a gradual and linear fashion over the post-intervention study period (*R*^2^ = 0.97, Fig. 3), and at 6 weeks post-intervention, total weight decreased by 14.4% (*p* < 0.05). However, changes in serum lipids were variable (Fig. 4). Total cholesterol and high-density lipoprotein cholesterol decreased significantly, with total cholesterol levels 6 weeks post-intervention [80 mg/dL (15.4), Supplemental Table 2] improving to below pre-diet levels [142.2 mg/dL (13.1), Supplemental Table 1]. Although not statistically significant, low-density lipoprotein cholesterol and triglycerides trended favorably toward improvement post-intervention (Supplemental Table 2). Plasma adiponectin significantly increased, while plasma leptin significantly decreased post-intervention (Fig. 5, Supplemental Table 2). There was no significant change in fasting active GLP-1 levels, although an overall upward trend was observed (Supplemental Table 2).

## Discussion

This is the first study to evaluate the feasibility of the Magnamosis magnetic compression device in creating a limited caliber side-to-side jejunoileal partial bypass in a nonhuman primate model. The results of this preclinical pilot study are highly encouraging. Device use (placement, detachment, and excretion) in the creation of a patent side-to-side jejunoileal anastomosis was occurred uneventfully in all animals demonstrating device feasibility. There were no device-related or anastomotic complications, indicating that use of the Magnamosis magnetic compression device to create a side-to-side jejunoileal anastomosis is also safe. Furthermore, successful delivery of the proximal magnetic anchor via upper endoscopy suggests that minimally invasive endoscopic device delivery is feasible and encourages further investigation of a completely endoscopic delivery approach. These results are consistent with prior experience regarding the safety of the Magnamosis device and feasibility of endoscopic device delivery [24–30].

Compared to other magnetic compression devices currently being studied for minimally invasive metabolic surgery, the Magnamosis device is distinguished by a shallow-fillet thick-wall ring design (interior area ratio of 0.006) with a polycarbonate tissue-compressing face [31, 32] (Supplemental Table 3). Magnetic compression anastomosis in the GI tract has been reported since the 1980s, with diverse applications including ileostomy undiversion, restoration of bowel continuity after urinary reconstruction, and duodeno-ileostomy accompanying sleeve gastrectomy for weight loss [24–30, 33, 34]. Recent experience with magnetic compression devices for esophageal atresia repair indicates that design differences can have significant effects on patient outcomes. As a notable example, clinical experience with two different magnetic compression devices has demonstrated that disc-shaped 50-mm^2^ tissue-compressing faces appear protective against post-anastomotic stricture, whereas disc-shaped 7-mm^2^ tissue-compressing faces appear to be associated with a high rate of dilation-refractory strictures [35, 36]. While a variety of magnetic compression devices are likely to be viable for future minimally invasive metabolic surgery, we urge rigorous reporting of device designs, including the compressive force at a reference of 3-mm face separation (*F*_R3_) and the corresponding spatially-averaged pressure on an interposed double-layer of bowel tissue (*P*_R3_) to facilitate informed comparisons prior to widespread adoption.

To our knowledge, this is the first study to evaluate limited caliber side-to-side jejunoileal partial bypass in a nonhuman primate model. As this nonhuman primate model provides preclinical fidelity in studying glucometabolic outcomes in humans by allowing tight control over experimental parameters (e.g., pre-intervention and post-intervention diet), the results of this study also represents a significant contribution to the understanding of the metabolic effects of ileal partial diversion. With the Magnamosis device, metabolic outcomes suggest limited-caliber jejunoileal partial diversion with a 15.5-mm anastomosis results in brisk onset of potent glucoregulatory effects, with rapid improvement of glucose homeostasis and resolution of systemic IR. The decrease in fasting plasma insulin and HOMA-IR to values below pre-diet baseline reflect resolution of compensatory pancreatic beta-cell insulin secretion and improved systemic IR, respectively [17]. This is consistent with established effects of conventional metabolic surgery observed independent of weight loss [37, 38]. Total weight loss of 14.4% confers significant metabolic benefit [39]. Furthermore, the gradual, consistent weight loss across the 6-week post-intervention period may mitigate malabsorptive sequelae or hypoglycemic episodes. However, although highly favorable, our findings likely underestimate the true metabolic impacts of the intervention because the observed metabolic effects were blunted by the high-sugar diet that was continued throughout the post-intervention study period. Thus, the potency of this intervention’s underlying physiologic mechanisms is likely more substantial than can be appreciated than our initial findings appreciated [3].

Despite the mixed results, likely due to the limited number of animals in the study, the observed effects on lipids are promising. Despite lack of statistical significance in this small sample size, the dramatic decrease in triglycerides reflects a clinically significant improvement in most animals. The considerable variability in the severity of hypertriglyceridemia between individual animals pre-intervention (68–1019 mg/dL) also likely skewed the analysis, resulting in a type II error. We suspect that the gradual trend toward improvement in low-density lipoprotein also reflects a clinically significant effect. In response to a change in nutritional status, lipid homeostasis (particularly lipoproteins) may remain in flux for 3 months before reaching a new setpoint. The 6-week study period was likely too short to appreciate the full impact of this intervention on lipid metabolism [18]. The increase in plasma adiponectin and decrease in plasma leptin levels were consistent with the expected effects of weight loss and parallel the described weight loss-dependent benefits of metabolic surgery [40]. In fact, the increase in plasma adiponectin to levels higher than those observed prior to initiating the diabetogenic diet is greater than would be expected from the observed degree of weight loss alone [41]. This likely reflects additional underlying beneficial effects of the intervention on plasma adiponectin that are independent of weight loss.

While we observed an overall increasing trend in circulating GLP-1 post-intervention, this ultimately did not reach significance. This may also be limited by the small sample size in this study which may be underpowered to detect a true difference. In addition, although HOMA-IR correlates with hyperinsulinemic-euglycemic clamp, considered by some to be the gold standard for assessing IR [42], glucose tolerance testing is required to appreciate the full effects of metabolic surgery on pancreatic beta-cell function and systemic insulin sensitivity [3]. Thus, the lack of glucose tolerance testing at regular intervals may have also limited our evaluation of this intervention’s impact on the post-prandial GLP-1 response. However, prior studies have focused on the robust GLP-1 response observed with direct enteral stimulation of the distal small intestine, as well as the subsequent significant improvement in insulin sensitivity and pancreatic beta-cell responsiveness [43–45]. GLP-1 receptor agonists have stimulated a frenzy of rapid pharmacologic investigation and surgical procedures that increase delivery of bile directly to the distal gut are considered effective strategies to reproduce some of these metabolic benefits, as is observed after Roux-en-Y gastric bypass [46–49]. As the Magnamosis device utilizes a narrow-caliber (15.5-mm outer diameter) partial bypass, diverting only a small volume of chyme to the distal ileum may capitalize on the metabolic benefits of distal enteral stimulation, while avoiding the sequelae related to malnutrition, bacterial overgrowth, and micronutrient deficiencies often observed with conventional metabolic surgery [10, 50]. However, additional long-term preclinical data is needed to validate the efficacy and durability of this approach.

Several limitations of this preclinical pilot study must be considered. Due to ethical considerations regarding not only large animal studies, but also primate-specific experimentation, a small sample size was chosen to study the a priori objectives of evaluating device feasibility and safety. Thus, this study is underpowered and not primarily intended to compare post-operative morbidity or long-term metabolic outcomes. However, in order to demonstrate the utility of this intervention with the Magnamosis device, animals served as internal controls and were compared pre- versus post-intervention. In addition, the small sample size limits the generalizability of our findings, as the variability between individual animals could have skewed data analysis. Further investigation is ongoing to expand the preclinical cohort and validate the results suggested by our findings. The 6-week post-intervention study period also limited our ability to appreciate the full effect on lipid metabolism, and to comment on the durability of a partial jejunoileal bypass created with the Magnamosis device. A longer study period will be required to fully delineate the metabolic effects and long-term anastomotic outcomes in future studies. Additional areas currently under further investigation include optimization of device placement (e.g., completely endoscopic approach) and device size for this minimally invasive metabolic surgery approach.

In conclusion, creation of a side-to-side jejunoileal anastomosis using a Magnamosis magnetic compression device is safe and feasible, but further optimization of minimally invasive device delivery is ongoing. While our results are promising, further investigations are ongoing to validate the metabolic efficacy of jejunoileal partial bypass and long-term durability of this treatment approach.

### Supplementary Information

Below is the link to the electronic supplementary material.

Supplementary file1 (DOCX 15 KB)

Supplementary file2 (DOCX 17 KB)

Supplementary file3 (DOCX 27 KB)

## Data Availability

Data available upon reasonable request to the corresponding author.
