# Supplementary material for: Evaluation of a Magnetic Compression Anastomosis for Jejunoileal Partial Diversion in Rhesus Macaques
Source: Obes Surg. 2023 Dec 23;34(2):515–23. doi: 10.1007/s11695-023-07012-4 (PMC10810932; doi:10.1007/s11695-023-07012-4)
Supplement: Supplementary file 1 — Supplementary file1 (DOCX 15 KB) [file 11695_2023_7012_MOESM1_ESM.docx]

**Supplemental Table 1 –** Serum metabolic parameters before starting the diabetogenic diet and pre-intervention (week 0) after 8 weeks of the diet.

|  | **Pre-diet** | **Week 0** |
| --- | --- | --- |
| *Insulin and Glucose* | | |
| FPI, µU/mL | 56.5 (13.2) | 118.6 (21.5) |
| HOMA-IR | 12.4 (2.5) | 27.5 (6.0) |
| FPG, mmol/L | 5.2 (0.3) | 5.1 (0.7) |
| *Lipids* | | |
| TC (mg/dL) | 141.4 (11.9) | 142.2 (13.1) |
| HDL-c (mg/dL) | 58.9 (7.8) | 53.8 (9.2) |
| LDL-c (mg/dL) | 68.9 (7.9) | 51.3 (5.9) |
| TG (mg/dL) | 79.5 (21.3) | 363.2 (178.0) |
| *Metabolic Hormones* | | |
| Adiponectin (µg/mL) | 8.7 (3.2) | 4.4 (1.7) |
| Leptin (ng/mL) | 23.0 (3.9) | 57.71 (3.2) |

Data reported as mean (SEM). FPG (fasting plasma glucose), FPI (fasting plasma insulin), HOMA-IR (homeostatic model assessment of insulin resistance), TC (total cholesterol), HDL-c (high-density lipoprotein cholesterol), LDL-c (low-density lipoprotein cholesterol), TG (triglycerides).
