# Supplementary material for: Evaluation of a Magnetic Compression Anastomosis for Jejunoileal Partial Diversion in Rhesus Macaques
Source: Obes Surg. 2023 Dec 23;34(2):515–23. doi: 10.1007/s11695-023-07012-4 (PMC10810932; doi:10.1007/s11695-023-07012-4)
Supplement: Supplementary file 2 — Supplementary file2 (DOCX 17 KB) [file 11695_2023_7012_MOESM2_ESM.docx]

**Supplemental Table 2 –** Serum metabolic parameters at 3 and 6 weeks after intervention compared to pre-intervention baseline (week 0).

|  | **Week 0** | **Week 3** | **Week 6** | **p-value** |
| --- | --- | --- | --- | --- |
| *Glucose and Insulin* | | | | |
| FPI, µU/mL | 118.6 (21.5) | 59.1 (20.1) | 43.1 (6.5) | 0.022 |
| HOMA-IR | 27.5 (6.0) | 10.8 (3.5) | 8.5 (1.4) | 0.022 |
| FPG, mmol/L | 5.1 (0.7) | 4.1 (0.2) | 4.4 (0.2) | 0.368 |
| *Lipids* | | | | |
| TC (mg/dL) | 142.2 (13.1) | 97.5 (7.8) | 80.0 (15.4) | 0.015 |
| HDL-c (mg/dL) | 53.8 (9.2) | 35.0 (5.5) | 32.0 (8.8) | 0.015 |
| LDL-c (mg/dL) | 51.3 (5.9) | 47.9 (2.7) | 34.1 (7.9) | 0.247 |
| TG (mg/dL) | 363.2 (178.0) | 135.2 (29.3) | 105.7 (13.9) | 0.165 |
| *Metabolic Hormones* | | | | |
| Adiponectin (µg/mL) | 4.4 (1.7) | 10.6 (3.9) | 13.5 (3.5) | 0.022 |
| Leptin (ng/mL) | 57.7 (3.2) | 37.6 (5.5) | 34.5 (7.2) | 0.015 |
| Fasting Active GLP-1 (pg/mL) | 1.8 (0.2) | 3.1 (0.6) | 4.1 (1.2) | 0.247 |

Data reported as mean (SEM). FPI (fasting plasma insulin), HOMA-IR (homeostatic model assessment of insulin resistance), FPG (fasting plasma glucose), TC (total cholesterol), HDL-c (high-density lipoprotein cholesterol), LDL-c (low-density lipoprotein cholesterol), TG (triglycerides), GLP-1 (glucagon-like peptide-1).
