# Supplementary material for: Evaluation of a Magnetic Compression Anastomosis for Jejunoileal Partial Diversion in Rhesus Macaques
Source: Obes Surg. 2023 Dec 23;34(2):515–23. doi: 10.1007/s11695-023-07012-4 (PMC10810932; doi:10.1007/s11695-023-07012-4)
Supplement: Supplementary file 3 — Supplementary file3 (DOCX 27 KB) [file 11695_2023_7012_MOESM3_ESM.docx]

**Supplemental Table 3 –** Device descriptions of magnetic compression devices under investigation for gastrointestinal bypass.

| Device Design | Tissue-Compressing Face Geometry | Tissue-Contacting Face Material | Endoscopic delivery notes | Interior Area Ratio | References |
| --- | --- | --- | --- | --- | --- |
| Shallow-Fillet Thick-Wall Ring | 15.5mm outer diameter | Polycarbonate | Carried at front of scope | 0.99 | (1-4) |
| Thin-Wall Octagonal Ring | 25mm outer diameter | Gold and Parylene | Through-the scope (ring self-assembles from eight linked linear magnets) | 0.3 | (5-7) |
| Thin-Perimeter-Lip Rounded Rectangle | 9mm length, 6mm width, 3mm thickness | Titanium | Carried at front of scope | n/a | (8-11) |

**Supplemental Table 3 References:**

1. Gonzales KD, Douglas G, Pichakron KO, Kwiat DA, Gallardo SG, Encinas JL, et al. Magnamosis III: delivery of a magnetic compression anastomosis device using minimally invasive endoscopic techniques. J Pediatr Surg. 2012 Jun;47(6):1291-5. PubMed PMID: 22703808.

2. Jamshidi R, Stephenson JT, Clay JG, Pichakron KO, Harrison MR. Magnamosis: magnetic compression anastomosis with comparison to suture and staple techniques. J Pediatr Surg. 2009 Jan;44(1):222-8. PubMed PMID: 19159747.

3. Pichakron KO, Jelin EB, Hirose S, Curran PF, Jamshidi R, Stephenson JT, et al. Magnamosis II: Magnetic compression anastomosis for minimally invasive gastrojejunostomy and jejunojejunostomy. J Am Coll Surg. 2011 Jan;212(1):42-9. PubMed PMID: 21184956.

4. Wall J, Diana M, Leroy J, Deruijter V, Gonzales KD, Lindner V, et al. MAGNAMOSIS IV: magnetic compression anastomosis for minimally invasive colorectal surgery. Endoscopy. 2013 Aug;45(8):643-8. PubMed PMID: 23807805. Epub 20130627.

5. Ore AS, Askenasy E, Ryou M, Baldwin T, Thompson CC, Messaris E. Evaluation of sutureless anastomosis after ileostomy takedown using the self-forming magnet anastomosis system in a porcine model. Surg Endosc. 2022 Oct;36(10):7664-72. PubMed PMID: 35157121. Epub 20220214. eng.

6. Ore AS, Ryou M, Messaris E. Sutureless laparoscopic intracorporeal ileocolic anastomosis using self-forming magnets. Tech Coloproctol. 2023 Dec;27(12):1379-80. PubMed PMID: 37594603. Epub 20230818. eng.

7. Gumustop D, Seddon D, Gumustop B, Wang J. A comparison of anastomosis strength between sutures, staples, and self-forming magnets. iGIE. 2022 2022/12/01/;1(1):11-4.

8. Gagner M. Side-to-side duodeno-colic anastomosis provides dramatic weight loss. A potentially strong anti-diabetic operation for type-2 diabetes. Minerva Chir. 2017 Jun;72(3):169-77. PubMed PMID: 28198178. Epub 20170214. eng.

9. Gagner M. Safety and efficacy of a side-to-side duodeno-ileal anastomosis for weight loss and type-2 diabetes: duodenal bipartition, a novel metabolic surgery procedure. Ann Surg Innov Res. 2015;9:6. PubMed PMID: 26473004. Pubmed Central PMCID: PMC4607140. Epub 20151014. eng.

10. Gagner M, Abuladze D, Koiava L, Buchwald JN, Van Sante N, Krinke T. First-in-Human Side-to-Side Magnetic Compression Duodeno-ileostomy with the Magnet Anastomosis System. Obes Surg. 2023 Aug;33(8):2282-92. PubMed PMID: 37393568. Pubmed Central PMCID: PMC10345004. Epub 20230702. eng.

11. Gagner M, Krinke T, Lapointe-Gagner M, Buchwald JN. Side-to-side duodeno-ileal magnetic compression anastomosis: design and feasibility of a novel device in a porcine model. Surg Endosc. 2023 Aug;37(8):6197-207. PubMed PMID: 37170025. Epub 20230511. eng.
